# ‘Perspectives on healthcare, chronic non-communicable disease and healthworlds in an urban and rural setting’ by Daniel Lopes Ibanez-Gonzalez

**DOI:** 10.3402/gha.v7.25867

**Published:** 2014-09-24

**Authors:** Catherine E. Draper

**Affiliations:** 1 MRC/Wits Developmental Pathways for Health Research Unit, Johannesburg, South Africa;; 2 UCT/MRC Research Unit for Exercise Science and Sports Medicine, Cape Town, South Africa

The burden of non-communicable diseases (NCDs) is increasing in South Africa in both urban and rural settings (1). Owing to the strain this growing burden places on the country's public health system, it is essential for policy-makers, researchers, and practitioners to adopt innovative approaches to understand healthcare access and experiences of those with these diseases in South Africa. Despite extensive healthcare system reforms in the 20 years since this country's first democratic election, a number of challenges persist, and much still needs to be done to provide equitable access to healthcare, and for the primary health care approach to be fully realised.

In his paper, ‘Perspectives on healthcare, chronic non-communicable disease and healthworlds in an urban and rural setting’ (2), Dr. Daniel Lopes Ibanez-Gonzalez acknowledges these healthcare challenges, and draws on concepts from objectivist and social constructionist approaches within public healthcare in order to expand our understanding of NCDs and healthcare access. One of the key concepts he uses is the ‘healthworld’, which is a ‘tool to explain the empirical complexity of health beliefs and behaviours’ (3).

Dr. Lopes Ibanez-Gonzalez's work was conducted in two contrasting study sites in South Africa, each with their own rich socio-political history: urban Soweto, Johannesburg, and rural Agincourt, a sub-district of the Bushbuckridge district in Mpumalanga province (north east region of South Africa). Although these two sites were found to be similar in terms of access to healthcare facilities and basic infrastructure, key differences exist in terms of population density (close to 30 times higher in Soweto (4, 5)), proximity to economic opportunities (in Agincourt, approximately 60% of men and increasing numbers of women migrate to more urban areas for work (6)), as well as access to certain basic amenities, such as running water in homes.

The contextual quantitative data presented in Dr. Lopes Ibanez-Gonzalez's paper indicated other similarities between Soweto and Agincourt in terms of low socioeconomic status, a high prevalence of NCDs, and relatively low healthcare utilisation amongst participants. His qualitative findings, from interviews with women with NCDs, confirmed these similarities with respect to low healthcare utilisation, and highlighted similar healthcare beliefs amongst participants from both settings. These findings also revealed that formal healthcare services were used by a small portion of individuals, although the quantitative data from Soweto showed an increased reliance on formal healthcare systems.

Regarding the low levels of healthcare utilisation in both these settings, it is tempting to focus on why women with NCDs are not using formal healthcare for the management of their condition/s. This could lead us towards a critique of urban and rural public healthcare, and there are undoubtedly a number of possible system-level factors that could go some way to explain poor utilisation. However, I believe that Dr. Lopes Ibanez-Gonzalez's study encourages another route of enquiry. His findings highlight the need to focus more on how individuals with NCDs talk about their illness and the ways in which they should cope with it. Interestingly, participants in his study placed less emphasis on availability, affordability, and acceptability of formal healthcare services. While these issues should not be ignored, Dr. Lopes Ibanez-Gonzalez has given prominence to the individual stories and perspectives of those living with NCDs, and his study points us towards finding a middle ground between hermeneutic and objectivist approaches to healthcare, by linking evidence-based medicine and narrative approaches (7).

Dr. Lopes Ibanez-Gonzalez points out that narrative approaches are already incorporated into the work of community-based organisations and traditional healers in African settings, making such approaches particularly relevant in the South African context. Furthermore, current healthcare discourse in South Africa around revitalising and re-engineering primary health care relies on the interface between formal healthcare and community-based services. As Dr. Lopes Ibanez-Gonzalez notes, a coherent lifeworld/healthworld schema, in which the construction of body narratives is central, could be especially helpful in the process of aligning formal healthcare with the needs of the communities they serve, and in the development and implementation of community-based interventions that are designed to address community healthcare needs.

Dr. Lopes Ibanez-Gonzalez concludes by referring to his study as the first step towards the realisation of an integrated healthcare approach focused on lifeworld/healthworld rationalisation and the on-going development of body narratives. With South Africa facing a quadruple burden of disease (1), and the applicability of such an approach to conditions other than NCDs, we surely need innovative perspectives such as this to take many more steps in the right direction.
